# Quantifying animal movement for caching foragers: the path identification index (PII) and cougars, *Puma concolor*

**DOI:** 10.1186/s40462-017-0115-z

**Published:** 2017-11-23

**Authors:** Kirsten E. Ironside, David J. Mattson, Tad Theimer, Brian Jansen, Brandon Holton, Terence Arundel, Michael Peters, Joseph O. Sexton, Thomas C. Edwards

**Affiliations:** 1 U.S. Geological Survey, Southwest Biological Science Center, 2255 N. Gemini Dr, Flagstaff, AZ 86001 USA; 2 Biological Sciences Department, Northern Arizona University, Flagstaff, AZ 86011 USA; 3 National Park Service, Grand Canyon National Park, Science and Resource Center, Grand Canyon, AZ 86023 USA; 4 Pterylae Systems, P.O. Box 35172, Phoenix, AZ 85069 USA; 5 Global Land Cover Facility, Department of Geographical Sciences, University of Maryland, 4231 Hartwick Road, College Park, MD 20742 USA; 6 U.S. Geological Survey, Utah Cooperative Fish and Wildlife Research Unit, Utah State University, 5230 Old Main Hill, Logan, UT 84322-5230 USA; 7 Department of Wildland Resources, Utah State University, 5230 Old Main Hill, Logan, UT 84322-5230 USA

**Keywords:** Behavior, Caching, Denning, Foraging, GPS telemetry, Mountain lions, Movement index, Path analysis, *Puma concolor*, *Statistical nonstationarity*

## Abstract

**Background:**

Many studies of animal movement have focused on directed versus area-restricted movement, which rely on correlations between step-length and turn-angles and on stationarity through time to define behavioral states. Although these approaches might apply well to grazing in patchy landscapes, species that either feed for short periods on large, concentrated food sources or cache food exhibit movements that are difficult to model using the traditional metrics of turn-angle and step-length alone.

**Results:**

We used GPS telemetry collected from a prey-caching predator, the cougar (*Puma concolor, Linnaeus*), to test whether combining metrics of site recursion, spatiotemporal clustering, speed, and turning into an index of movement using partial sums, improves the ability to identify caching behavior. The index was used to identify changes in movement characteristics over time and segment paths into behavioral classes. The identification of behaviors from the Path Identification Index (PII) was evaluated using field investigations of cougar activities at GPS locations. We tested for statistical stationarity across behaviors for use of topographic view-sheds. Changes in the frequency and duration of PII were useful for identifying seasonal activities such as migration, gestation, and denning. The comparison of field investigations of cougar activities to behavioral PII classes resulted in an overall classification accuracy of 81%.

**Conclusions:**

Changes in behaviors were reflected in cougars’ use of topographic view-sheds, resulting in statistical nonstationarity over time, and revealed important aspects of hunting behavior. Incorporating metrics of site recursion and spatiotemporal clustering revealed the temporal structure in movements of a caching forager. The movement index PII, shows promise for identifying behaviors in species that frequently return to specific locations such as food caches, watering holes, or dens, and highlights the potential role memory and cognitive abilities play in determining animal movements.

## Background

Why, when, and how organisms move through landscapes have fascinated biologists for generations, resulting in a substantial body of literature (for reviews see [1, 2]). Many initial studies of movement and behavior including, spatial learning [3], optimal foraging [4, 5], scaling of resource selection [6], navigation [7], and spatial cognition [8], demonstrated the potential complexity of animal movements. Yet random search behaviors have been studied extensively [9–12] and the importance of memory in driving animal movement remains a major challenge [13]. Fueling a growing field of study, animal movements measured via GPS telemetry provide information about animal behavior that cannot be observed directly for many species. Improving our ability to interpret potential motivations behind observed movements in GPS telemetry data will provide a deeper understanding of complex movement phenomena, such as caching, migration, and home range use [14]. This includes improving detection of the use of spatial memory, which increases efficiency of access to predictable food sources or caches, nesting or denning locations, safe resting sites, and hiding sites for dependent young. Long-term memory aids navigation of complex landscapes to re-locate rarely used, but essential resources (e.g. hunting grounds, calving grounds, nesting beaches, or movement and migration corridors).

The frequency and regularity of revisits are important metrics to consider when studying animal movements and can aid in assessing habitat use at different temporal and spatial scales. Identifying areas of home ranges or utilization distributions that are frequently revisited have been addressed in only a few studies [15, 16], but these have shown that including a temporal component can dramatically change how use of space is perceived. Residency time—the length of time spent at a particular site—can reflect the value of a particular site to an animal [17], and identifying return points and frequency of site recursion [18] have been proposed for detection of memory processes in animal movement data [13]. When the spatial scale of movements is large, it is likely that movement recursions are memory-driven because re-visited areas are beyond an animal’s perceptual range; recursion thus serves as a gauge for the use of spatial memory, navigation ability, and cognition [15]. Likewise, precise movements (i.e., with low navigational variance) may also be strong indicators of memory-guided movement processes [13].

Simulating animal movements has employed a variety of approaches, including correlated random walks, Brownian motion, Levy walks, least-cost path, resistance surfaces, state-space, and Markov chain modeling frameworks. These methods all require a large number of assumptions, which may or may not represent real animal movement characteristics. Although state-space modeling can recreate many characteristics of animal movement, it might be most useful for identifying inconsistencies in our understanding of animal movement, with the frequency of reversals being one of them [19]. Small changes in the specifications of distributions for net-displacement [20] and turn-angles [19] can have large consequences on simulated movements. Similarly, identification of search modes, issues of spatial scale, and the resolution of temporal sampling have led to controversy on parametrization of searching [9]. Popular techniques for identifying movement corridors, least-cost path, and circuit theory resistance surfaces are sensitive to scale and behavioral state [21] and have been shown to misrepresent animals’ use of landscapes when compared with observed movement data [22].

Many current approaches to analyzing animal movement base probability distributions, dwell times, and degrees of diffusion in random walk frameworks, on broad-scale, seasonal or annual home-range use, resulting in controversy regarding model misspecifications [9, 12, 20, 23]. As a result, some researchers are advocating movement ecologists trade this “top-down” approach for a more “bottom-up” approach, in which analysis begins at path segments, the finest level of observation. In an effort to improve understanding of the role of environmental cues in animal movement, and recognizing that behavioral states are important conditions of resource selection, more attention is being given to statistical stationarity (the stability of statistical parameters) [21, 23–25]. Recent studies have shown many advantages using metrics of path segments such as path straightness [26], entropy of path tortuosity [27], and autocorrelation [24, 28]. Yet the segmentation of paths is often arbitrarily set, and how best to segment paths remains an open question.

Foraging success is an important determinant of fitness and likely explains a large portion of movement behavior*.* Past studies have focused on the movements of species whose foraging habits reflect those of “grazers”—i.e., species that search for small, patchily dispersed food items, requiring a large amount of time to be spent in searching and gathering activities. Examples of this are seals foraging for fish, bees collecting pollen, or birds feeding on insects. Much of the early work on animal movements focused on organisms that reflect this foraging strategy [29], and many early terrestrial GPS telemetry studies were applied to herbivorous grazers, e.g. elk [30, 31] and bison [32], resulting in grazing strategies shaping the development of many of the analytical approaches in place today. Similarly for marine GPS telemetry applications, initial analytical approaches were shaped by littoral and pelagic grazers, e.g. sea turtles and seals [33–35].

Movements of species that forage by periodically obtaining or visiting relatively large, concentrated food sources are often analyzed in the same manner as other foragers. However, many predators (e.g., large felids, wolves, bears, and meso-carnivores) can take prey items several times their size. Subsequent caching and returns to feed over a period of time result in movements that can differ in nature to those that are best studied using correlated random walk/searching behavior. These concentrated food sources are often revisited over long periods of time, during which caching and hoarding helps maximize an individual’s use of the food source [36].

Caching is not limited to predators, but has been most extensively studied in rodents and birds [37]. Many of these studies have been either experiments to test the cognitive and navigational abilities of these organisms or from direct observation, unlike the indirect observations and uncontrolled environment in relocation studies (e.g. repeat observations of study animal locations from radio or GPS telemetry, or tracking). The characteristic movement of this group of foragers is site recursion. Often organisms that forage this way leave the food item for periods of time to perform other activities (e.g., rest, hydrating, care for offspring) and return later to feed again. Behavioral studies have revealed strong memory and cognitive abilities in these types of foragers [38]. This foraging strategy can result in a greater portion of time spent on resting and/or traveling between hunting grounds compared to the searching that has been the focus of movement ecology. Spatial cognition, navigation ability, memory, and learning likely play an important role in the movement of these foragers, thereby requiring a high degree of model sophistication in terms of multiple movement modes, several static and dynamic landscape covariates, and conditional use to adequately specify and simulate movements on real landscapes [39].

Over larger spatio-temporal scales, site recursion can be a movement characteristic of many animals, not only caching foragers. Central place foraging, where animals return repeatedly from foraging to a central location such as a nest, burrow, or resting site, has been documented in many animals from insects [40], to birds [41], to monkeys [42]. Likewise, site recursion can be common for many animals using a limited resource, such as water sources in arid ecosystems [43]. Seasonal migrations are yet another example of a large scale spatio-temporal pattern where species revisit profitable places [44].

Here we propose and explore an analytical framework incorporating findings from several recent studies on path-level analysis for measuring and interpreting animal movements into behaviors at multiple scales. We develop an index, the Path Identification Index (PII), to quantify characteristics of movement related to daily circadian activity levels and foraging behavior, as well as seasonal changes in movements related to reproductive status and food availability. We test the analytical framework using GPS telemetry data collected from a food caching predator, the cougar (*Puma concolor*, *Linnaeus*) in the southwestern United States. Assuming resource selection changes with changes in behavior and across different life stages, statistical stationarity is evaluated for circadian and foraging activities in cougars of different sexes at different life stages: territorial males, a non-reproductive female, denning females, and a female accompanied by sub-adult offspring. We also test our method of movement-pattern identification and path segmentation with in situ investigations of cougar activity.

## Methods

### Study area and data collection

The study region is characterized by steep elevation gradients and complex terrain that results in inter-digitation of a variety of biomes and vegetation communities. Climate is variable but generally considered semi-arid. Precipitation occurs as snow in winter for a large portion of the ranges of the cougars included here, but a portion of the study area experiences a bimodal precipitation regime from a summer monsoon contributing a significant portion of total annual rainfall. Mule deer (*Odocoileus hemionus*, *Rafinesque*) and elk (*Cervus elaphus*, *Linnaeus*) are the primary prey but varied in availability across the six study animals.

### Southwest region of USA

Capture, GPS-collar tagging, and release of cougars has been conducted in northern Arizona and southern Utah and Nevada, USA, from 2003 to present by the U.S. Geological Survey and the National Park Service (Northern Arizona University IACUC Protocol # 02–082-R4), resulting in GPS-tracking and monitoring of 74 study animals thus far. Our analysis focused on a subset of six individuals that met three criteria: 1) few missing data (≥ 85% fix success rate), 2) long periods of continuous monitoring, with a minimum of 1000 observations, and 3) established home range with no evidence of dispersal (Table 1). Six cougars met the above criteria for analysis: four females and two males, ranging in age from 1.5–4 years in age. Two females (C04 and AS02) denned during observation, and one was known from field observation to be accompanied by two sub-adult offspring.

**Table 1:** Study animals selected for analysis

Cougar ID		Sex	Age (yrs)	FSR	# Obs.	Timespan	Notes
1	C04	Female	2	89%	1682	8/4/2003–6/14/2004	Denned during observation, Primary diet = deer and elk
2	AS02	Female	1.5	85%	1886	10/2/2010–10/6/2011	Denned during observation, Primary diet = deer
3	P26	Female	3.5	93%	1730	10/27/2009–9/1/2010	With offspring during monitoring, Primary diet = deer and elk
4	C07	Female	3	91%	1121	12/14/2004–7/7/2005	No evidence of reproductive activity Primary diet = deer and elk
5	Z04	Male	2	85%	1356	1/9/2007–9/19/2007	Diet = unknown,
6	AS07	Male	4	91%	2882	7/5/2012–11/26/2013	Primary diet = deer

Cougars were fitted with Telonics (Mesa, AZ, USA) GEN3 or GEN4 GPS collars with the ARGOS satellite feature programmed to deliver GPS locations daily. Collars were programmed to acquire six locations a day, every four hours, resulting in two nocturnal locations at 9:00 pm and 1:00 am: two roughly crepuscular locations at 5:00 am and 5:00 pm and two diurnal locations at 9:00 am and 1:00 pm local time (Mountain Standard Time). The GPS fix-acquisition window was set to 3 min, allowing a battery life of approximately 12–14 months, after which we programmed automatic releases to drop collars. We recovered the collars for the six study animals and downloaded GPS data directly, replacing the ARGOS transmitted data. Collars did not include activity sensors. Field investigations of GPS locations were conducted to document cougar prey items and activities [45].

#### Movement metrics

**Fig. 1:**
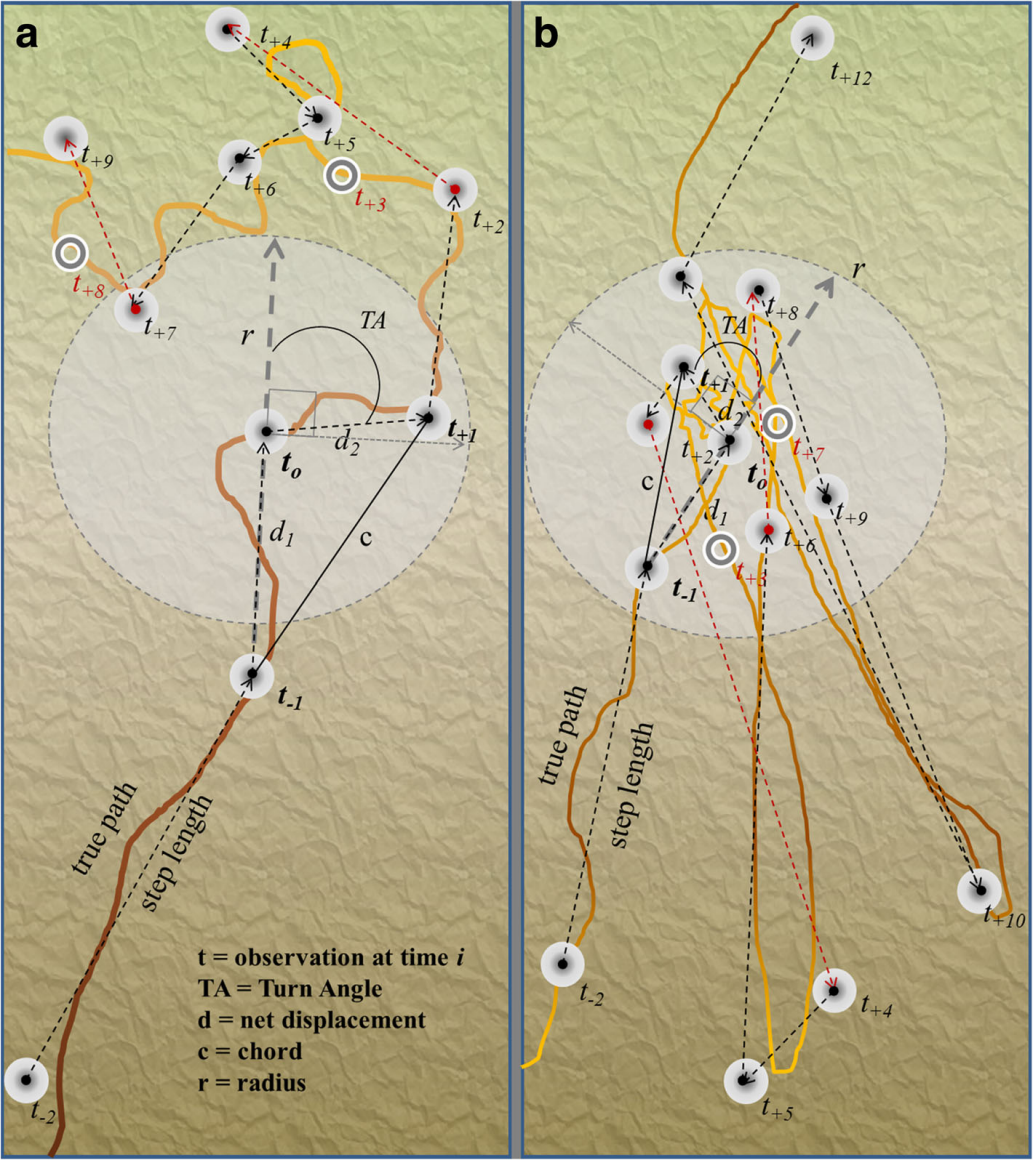
Summary of derived metrics used in wildlife GPS telemetry and simulated movement studies. Common movement metrics derived from point observations along an animal’s path include step-length, the Euclidean distance between consecutive points in time, which is easily converted to speed by dividing the net displacement distance by the associated time interval. Turn-angle is another common measure in the movement ecology literature. Less frequently used measures include path tortuosity or straightness that compares the net displacement over a series of observations (the chord) to the length of the path. Residency times or re-visitation rates are commonly estimated using a radius around an observation to measure the number of observations within a given distance for a given time interval. **a.** An illustrated movement type that has been the focus of movement ecology literature, a correlated random walk in response to a landscape covariate. Slow speed combined with high degrees of turning (search behavior shown in orange) in high quality habitat (green background) compared to fast directed movements in low resource availability (tan background) areas have dominated the literature. **b.** Illustrated movement path of caching or hoarding foraging movements where feeding is interrupted by short periods of other activities, such as resting away from the cache. The illustration shows speed and turn-angle are not always associated with foraging, but site recursion and how observations are related to one another in space and time show a movement pattern. Open circles represent failed fix attempts and offset location positions are used to illustrate the imperfect detection of GPS telemetry

We explored 4 characteristics of movement and analyzed their contribution to describing cougar movement behaviors using cross- and temporal autocorrelation. We define a movement as the mean difference in location between a time (t_0_) and those preceding (t_−1_) and following (t_+1_) (Fig. 1).

#### Speed

The Euclidean distance between consecutive locations divided by time between locations (meters per minute). We estimated speed of travel at *t*_*0*_ by treating it as an observation along a continuum, by averaging the net displacement (step-lengths, *d*_*1*_ and *d*_*2*_) divided by the time interval from the previous, *t*_*−1*_, and following, *t*_*+1*_, observations.

1 $$ {S}_{t_0}=\frac{\frac{d_1}{t_0-{t}_{-1}}+\frac{d_2}{t_{+1}-{t}_0}}{2} $$

#### Turning

Turn-angles (TA) associated with *t*_*0*_ were measured in degrees between *t*_*−1*_, *t*_*0*_, and *t*_*+1*_. Exploratory analyses of histograms showed little preference for turning towards the left or right for our study animals, so the absolute value of turn-angle was used.

2 $$ {TA}_{standard}=\mid {TA}_{degrees}\operatorname{}\mid /180\operatorname{} $$

#### Tortuosity

**Fig. 2:**
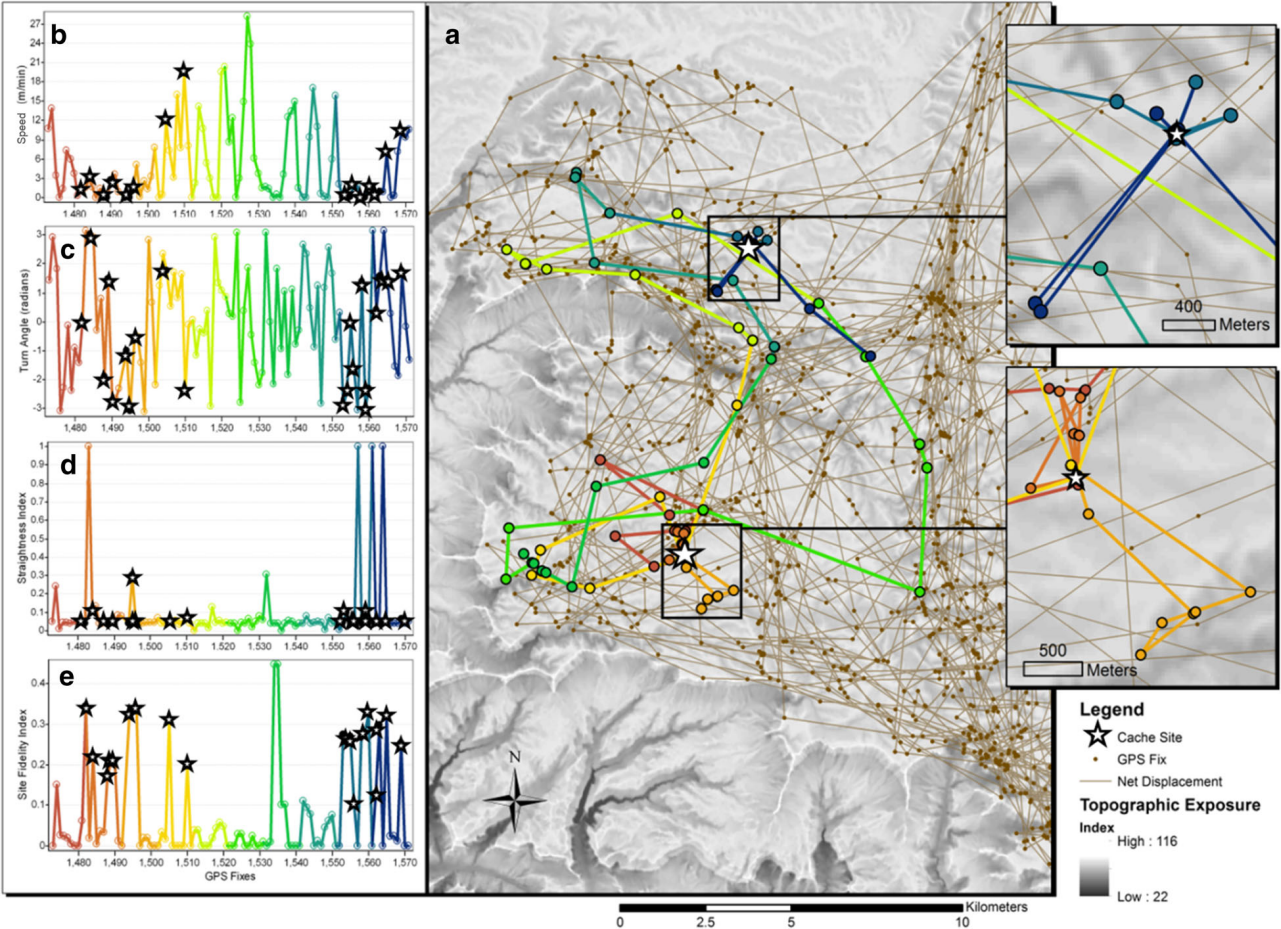
**a** A sixteen-day movement from March 7, 2013 – March 23, 2013 of an adult male cougar (AS07) used to illustrate patterns in speed, turning, straightness, and site fidelity with GPS points and paths color-coded from red to orange to yellow to green and then to blue through time. Insets of movements around known prey (mule deer) cache sites denoted by white stars demonstrate that it is common for cougars to intermittently feed on prey items, leave the cache site for periods of time, and return at a later time. **b** Graphs of speed from net displacement and **c** degree of turning alone demonstrate they are not always effective indicators of feeding behavior because associated measures of velocities can be both small and large and turning angle also can be highly variable, while the **d** straightness index captures the short bouts of leaving and returning to caches. **e** The site fidelity index captures when cougars are feeding at cache sites

Many measures have been proposed for measuring the tortuosity, or sinuosity (degree of turning) in a movement path [26, 27, 46]. We used the simplest measure, the straightness index at the finest temporal resolution of our data, which is the ratio of total distance traveled to net displacement (the chord). This index results in straight movements having low values and highly tortuous movements having high values. At a 4-h sampling window, the circadian feeding patterns of cougars often result in one or two observations where the cougar has left a cached prey item to rest and then returned to feed (Fig. 2d). These short bouts of site recursion were captured by the straightness index, using the distance of two consecutive step-lengths divided by the net displacement (the chord) Fig. 1.

3 $$ {M}_{straightness}=\left({d}_1+{d}_2\right)/c $$

#### Clustering

Identifying feeding behavior of cougars can be especially challenging because of their tendency to leave cache sites to bed some distance away and then return to the site to feed [47]. This can result in weak correlation between speed and turning (Table 2 and Fig. 2), which has been used to indicate foraging in many other species [30, 48].

**Table 2:** Pearson correlation coefficients (mean and range) between tortuosity (measured using the straightness index), estimated speed, absolute turn-angle, and site fidelity (spatio-temporal clustering) averaged across the six cougars described in Table 1

	Straightness Index	Speed	Turning
Speed	0.084 (−0.035–0.164)		
Turning	0.377 (0.325–0.426)	0.280 (0.143–0.373)	
Site Fidelity	0.001 (−0.035–0.043)	0.423 (0.386–0.458)	0.175 (0.114–0.237)

Cougar biologists have resorted to measures of spatial clustering in GPS locations to indicate kill/cache sites for dietary studies. These measures are similar to others found in the movement ecology literature such as residency time [17], first passage time [49], and fractal dimensions [50], wherein a moving spatial window/distance radius (Fig. 1) is used to measure how an observation relates to others in space and/or time [51]. We developed a similar measure, to which we refer as “*site fidelity”* that measures the clustering of observations within a given spatial window and is weighted by the length of time spent at that location. Two consecutive locations (8–24 h temporal windows for most GPS acquisition programs) within 100 m of one another are generally considered adequate to document cougar kill-cache sites [52]. Therefore we set our spatial circular window radius to 100 m around the location *t*_*0*_. Because we were also interested in distinguishing between the spatial clustering associated with den sites from that associated with kill sites, we chose a forward and backward temporal window of 8 weeks, the maximum potential time a den would be used before cougar cubs begin traveling with their mother. Observations outside of the designated spatial-temporal window were valued at zero for this index. The near tool in ArcGIS v10.2 Analyst toolbox was used to measure the distance (*d*) of each observation to every other observation in the dataset for an individual. The resulting distance table was imported into Microsoft Access, and SQL queries were used to select records within 100 m and +/−8 weeks of one another. The mean of the distances between selected observations was then multiplied by the fraction of observations out of the total possible for the given time period. This results in an index measuring the proximity of observations to one another weighted by the length of time that was spent at that site, but does not account for the potential effects of missed fixes (Fig. 1).

4 $$ {M}_{SiteFidelity}=w{\overline{d}}_{i,j} $$

where *w d* is the weighted average distance for observations within a defined temporal and spatial window (i,j). The weight is the number of *d*_*i,j*_ observations divided by the number of maximum possible for the temporal window.

#### Movement Index

Our approach is similar to the partial sums approach of Knell and Codling [51], in which step-length and turn-angle were standardized and inversely summed. They found this approach superior among a suite of methods for identifying behavioral switches between area-restricted searching and directed persistence. In addition to speed and turning, we also incorporated indices of straightness and site fidelity, which added valuable information for identifying caches and other frequently used locations. All four measures were rescaled to values between 0 and 1 by dividing by the maximum observed value for each individual. Net displacement values of the straightness index had some extremely high values, leading to a strongly skewed distribution. To adjust for this, we replaced the maximum with the 90th percentile. Values in the 90th–100th percentile were given the value of 1. The inverse of the scaled speed (1- *s*_*standard*_), was added to the three other standardized measures, resulting in the index having a potential range between 0 and 4. Low values of this composite index represent fast, straight, movements with large net displacement performed in areas not frequently visited; high values indicate slow, tortuous (both high degree of turning and little net displacement) movements in frequently visited areas.

Temporal autocorrelation in the movement index was then used to identify paths, defined here as a time-series of similar observations. The temporal lag found in the autocorrelation measures were then used to set a window size for a centered moving average of the movement index through time, resulting in the Path Identification Index (PII). The PII was then classified using user discretion into three classes: 1) directed movement – fast, straight movements associated with low index values, 2) search – movements with some tortuosity and mid-level net displacement, and 3) caching/denning – movements associated with high PII values. The classification of observations was then compared to previously collected data from field investigations (see Mattson et al. [45] for methods). Changes in the frequency of the movement behavior classes over time were used to identify large scale or seasonal shifts in animal movements.

Movement classes—directed, search, and cache—by time of day were used to explore the statistical stationarity (changes in resource selection by behavior) of movement behaviors. A step-selection approach for assessing resource use was employed, comparing paired use versus availability in a binary logistic regression [53]. For each observation at *t*_*−1*_, a buffer of radius equal to the maximum 4-h step-length observed for that study animal (*d*_*max*_) was set to define availability. Because our covariate of interest had a resolution of 30 m, and the distance a cougar is able to move in a 4-h period is several kilometers, we calculated the mean value across the window of availability (coded as 0) to compare to the value at location *t*_*0*_ (coded as 1). This resulted in a relative assessment of use compared to the average of what was available during each movement.

Observations for each study animal were subdivided based on circadian classification (nocturnal, crepuscular, and diurnal) and path classification (directed, search, and caching), resulting in 9 data subsets per animal. Logistic regression models were used to calculate beta (*β*) coefficients in Minitab v17 statistical software to assess the statistical stationarity of selection and the potential utility of the movement index for creating biologically relevant segmentations of movement paths.

Topographic indices are strong factors in resource-selection assessments of cougar habitat use [45, 54]. We explored a measure related to topographic exposure and the view-shed concept [55, 56], where terrain features such as ridgelines, canyon rims, and mountain tops with large view-sheds have high index values. Narrow valleys, draws, and canyon bottoms, all have small view-sheds and thus low index values of topographic exposure. Relatively flat areas have mid-level index values, of approximately 83, and slopes typically have values <80. We hypothesize the attraction of cougars to topographically complex landscapes is a function of seeking safe resting and denning sites (low topographic exposure and therefore low visibility) and also vantage points while detecting and pursuing prey (high topographic exposure and view-sheds). This hypothesis suggests statistical nonstationarity, dynamic movement, and selection of different ranges of topographic exposure for different behaviors.

## Results

**Fig. 3:**
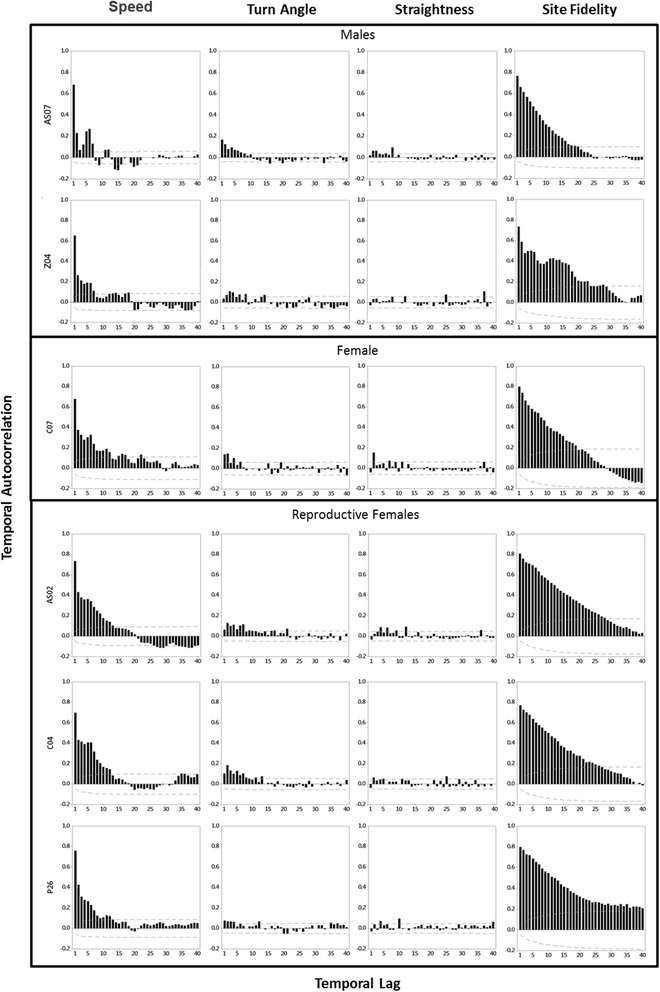
Temporal autocorrelation, shown with 5% significance limits (dashed lines), of movement metrics for each study animal. The temporal lag is equivalent to the fix interval of 4 h. All study animals showed some degree of autocorrelation in the speed of movements, though males (top two panels) less so than the females (bottom four panels). Very little autocorrelation occurs across all the study animals for turning and straightness of movements. The site fidelity measure succeeds in showing this strong temporal autocorrelation in movements

**Fig. 4:**
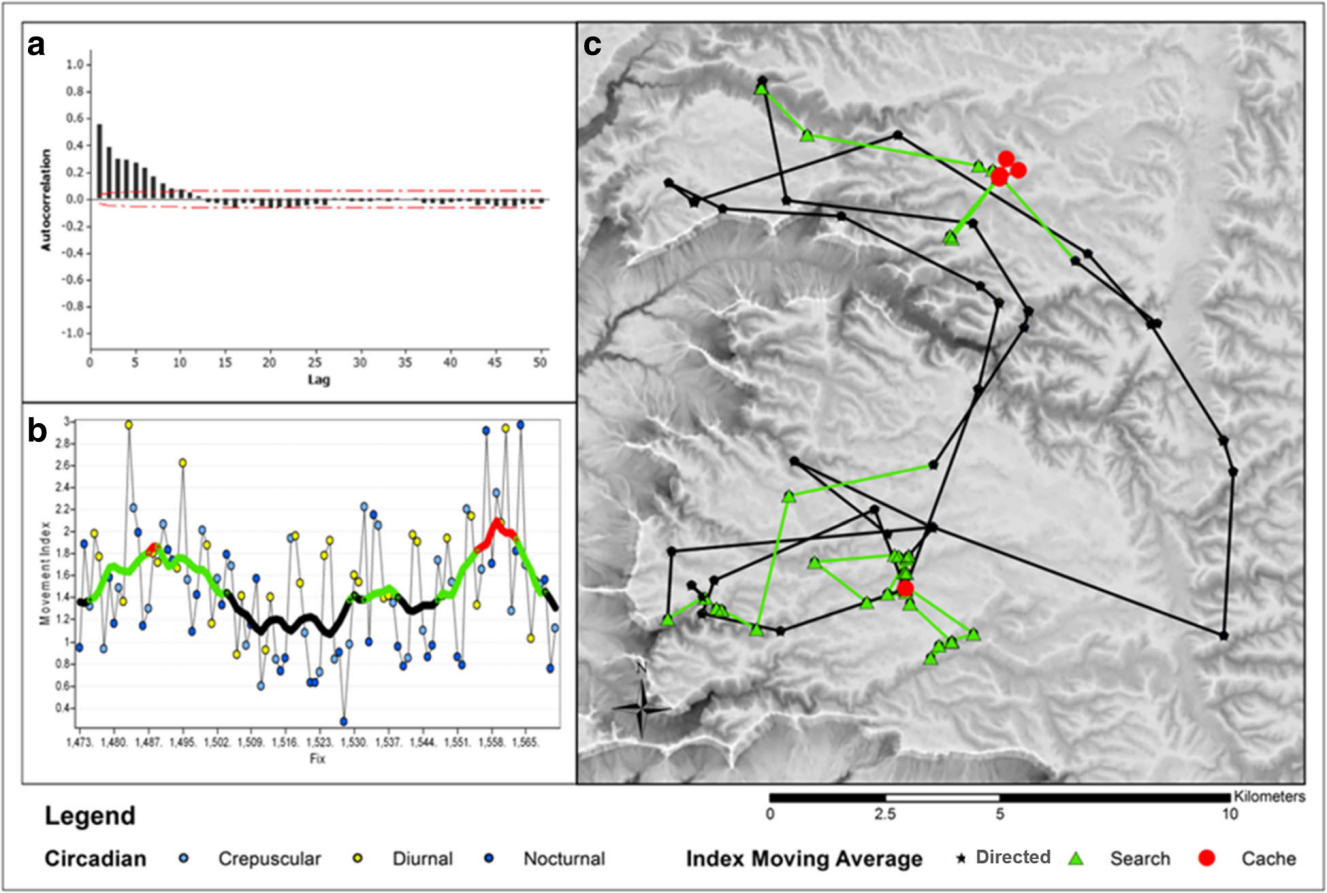
**a** Temporal autocorrelation of the movement index for AS07’s period of observation shows movement is correlated in time. The lag time, in number of consecutive observations, is approximately 12 observations on average before correlation reaches 0. The mean lag time across our 6 study animals was a 15-fix lag (~2.5 day period). **b** We used this lag period to set a 15-fix, centered moving average window to identify coarser scale movement patterns. Rapid directional movements which have a low index value were identified as directed movement between areas AS07 searched for prey. Mid-level index values (1.4–1.8) were identified as searching behavior due to changes in speed, straightness, and site fidelity. Movements associated with an index greater than 1.8 were classified as potential cache sites and movements associated with likely feeding behavior. The two cache sites shown below were verified in the field to be locations were AS07 cached and fed on mule deer carcasses. Search and feeding behaviors occurred at all times of the day. Directed movements (low PII values) are primarily conducted during nocturnal hours. **c** Same movement of AS07’s shown in Fig. 2, displayed as three classes of movement

None of the movement metrics showed strong correlation with one another, suggesting each measure contributes unique information on cougar movement. At a 4-h time interval, we found little correlation (0.28 +/− 0.15 SD on average) between turning and speed of movements (Table 2), and turning angle showed very little temporal autocorrelation (Fig. 3). The highest correlation coefficient for an individual was 0.458. Temporal autocorrelation was found over short periods of time in the speed and site fidelity of each study animal, though more so of females than males. Similar to turning, the straightness index did not show any patterns of temporal autocorrelation. The sum of the movement metrics into the composite movement index showed similar temporal autocorrelation across individuals, with an average temporal lag of 15 observations (equivalent to 2.5 days) (Fig. 4a). The 16-day movement of AS07 (Fig. 2), which encompassed two mule deer kills, illustrates common patterns in speed, turning, straightness, and site fidelity over time. Decreased speed did indicate the start of feeding behavior but was not a good indicator alone of the length of time spent at a kill site due to cougars leaving and returning to cached prey items as time progresses (inset maps in Fig. 2). Turn-angle was highly variable and independent of speed. The straightness index provides a good indication of site recursion, in this case when cougars leave caches to rest some distance away and return at a later time. The site fidelity metric provided a good indicator of when a cougar is at a cache site, the length of time spent at a cache site, and the periodicity of caching over time.

We used the temporal autocorrelation of the index to set a moving window to smooth the circadian rhythms in the various metrics to identify periodic patterns in movement. Smoothing the index using a centered moving average showed periodicity in the movement data related to foraging activities, and provided a means to identify paths (Fig. 4). Defining high PII values (>1.8) as caching behavior, and comparing with previously collected data on field visits of GPS clusters showed a high classification accuracy, with 75% of documented caches and 97% of non-cache related activities across all study animals being classified correctly. This resulted in an overall classification accuracy of 81% (*n* = 217) (Table 3). The highest classification accuracy was for C04, 92%, which had relatively equal sampling for caches (*n* = 26) and non-caching activities (*n* = 22). The lowest was for P26, a female known to be accompanied by two sub-adult offspring during monitoring, which had an overall classification accuracy of 70% (*n* = 67). The majority of misclassifications was due to cache sites being identified as searching (*n* = 18). This was likely due to having three individuals feeding on a kill, which resulted in shorter time spent at caches and suggests the PII threshold of 1.8 for identifying caches for females with sub-adult offspring could be lowered. Figure 4 shows directed movements were primarily conducted during crepuscular and nocturnal hours. Likewise, diurnal locations are more frequently associated with higher movement index values, suggesting a more sedentary level of activity during daylight hours.

**Table 3:** Comparison of PII classification of non-feeding (directed or searching) and feeding/cache sites with field verifications of those activities for five of six cougars described in Table 1

	Correct	Incorrect	Total
AS02			
Non feeding	2	0	2
Cache	20	4	24
Total	22	4	26
	85%		
C07			
Non feeding	26	0	26
Cache	17	10	27
Total	43	10	53
	81%		
AS07			
Non feeding	3	0	3
Cache	13	3	16
Total	16	3	19
	84%		
C04			
Non feeding	22	0	22
Cache	26	4	30
Total	48	4	52
	92%		
P26			
Non feeding	7	2	9
Cache	40	18	58
Total	47	20	67
	70%		
All			
Non feeding	60	2	62
Cache	116	39	155
Total	176	41	217
	81%		

Movements classified as non-feeding were considered correct if site investigations found no prey remains or signs of scavenging, and were considered incorrect if any of those evidences were present. Cache site classifications were considered correct if prey remains were found or evidence that the prey item had been scavenged, and as incorrect if no prey remains were found

**Fig. 5:**
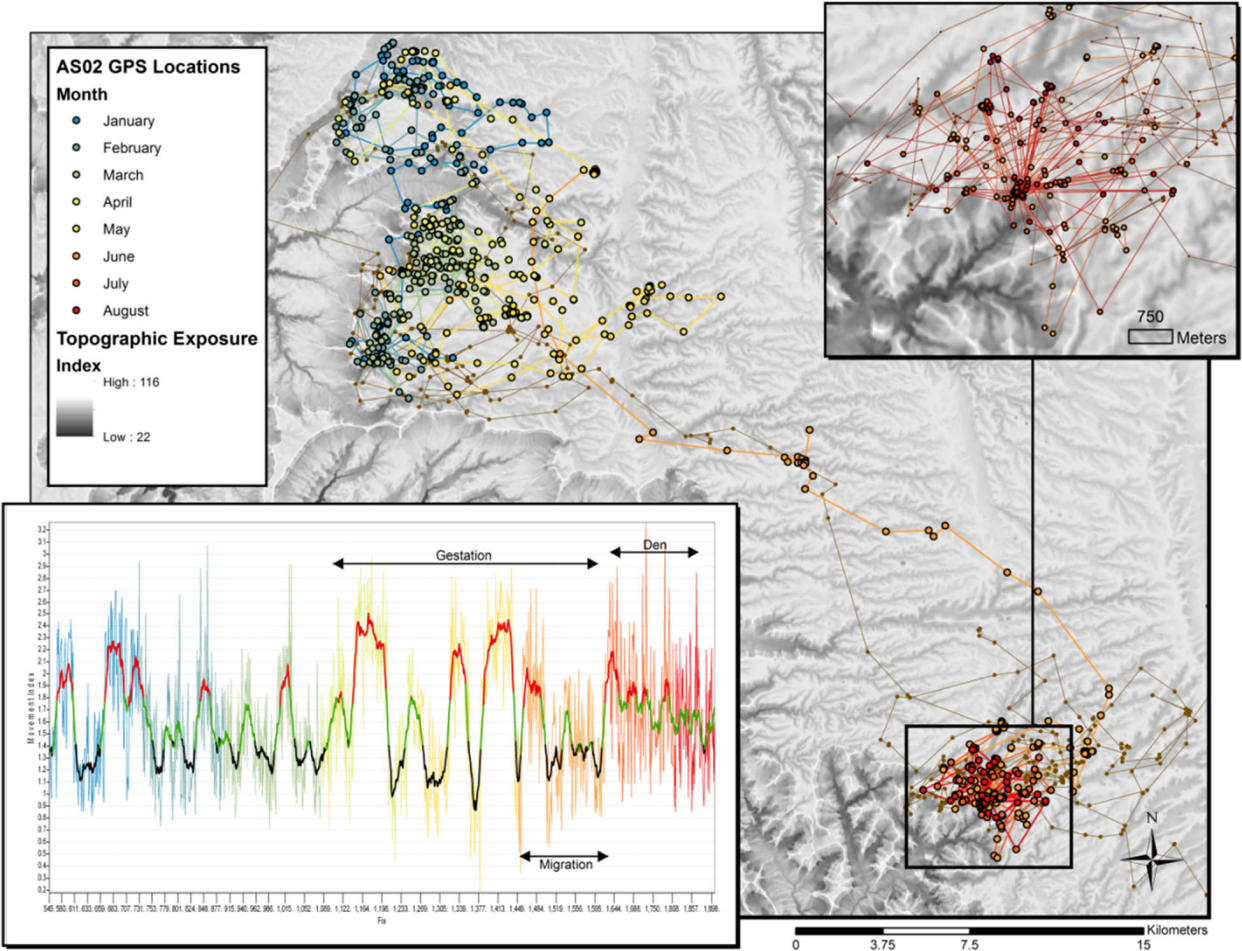
Patterns in the frequency of movement classes show broader seasonal signals in movement. Eight months of movements of a young female cougar occupying the North Rim of the Grand Canyon shows seasonal changes in the frequency of movement classes may be indicative of other behaviors with longer temporal windows. On June 29th, AS02 denned below the canyon rim and followed this with a bout of hunting and feeding near the den, returning frequently to the den site. The movement index, PII, signals this change in behavior by showing a period of time when directed movement did not occur. Prior to denning, AS02 moved to higher elevations on the Kaibab Plateau following migratory mule deer to their summer range. This period was marked by a longer and more frequent period of directed movement. Based upon the date of AS02’s first location at the den site, a ~90 day gestation period for *Puma concolor*, and the movement index, suggests AS02 spent longer at her kill sites during gestation than prior to pregnancy

Plotting PII over seasons showed temporal changes in the frequencies of behavioral classes, indicating changes in behavior for reproductive females and potential range shifts (Fig. 5). PII plotted over time shows the frequency of kills, duration of time spent at cache sites, and periods of directed movement. Prolonged periods of directed movement can be indicative of migration, as was the case for AS02 (Fig. 5) who followed migratory mule deer to their summer range at higher elevations on the Kaibab Plateau, Arizona. AS02’s period of denning below the rim of the Grand Canyon and foraging for mule deer above the rim was indicated in the PII by a lack of directed movement for a period of two months, and the highest PII values observed for the entire record, due to high values in site fidelity metric when at the den site and frequent recursions between caches and the den. Given AS02’s first location date at the den site and an average gestation period for cougars being 90 days shows the time spent at kill sites during gestation was prolonged compared to the period prior to conception, possibly reflecting changes in bioenergetic state.

Subdividing observations by circadian and PII class resulted in varying beta coefficients relating use versus availability of topographic exposure (Appendix). The range of topographic exposure was variable across the study animals, with the most complex terrain within Z04’s home range near Zion National Park, Utah, USA and AS02 and AS07s’ home ranges on the north rim of the Grand Canyon, Arizona, USA. The variability in topographic exposure was far less for C07 and C04, who inhabit a relatively flat area bisected by a series of narrow canyons south of Walnut Canyon National Monument, Arizona, USA. P26 inhabited the most moderate terrain of the six, occupying an area of small ridges and draws south of the Grand Canyon National Park, Arizona, USA. Due to differences in the availability of topographic exposure between home-ranges, and possibly differences in selection of exposure between individuals, the scaling and absolute values of the beta coefficients vary across individuals.

**Fig. 6:**
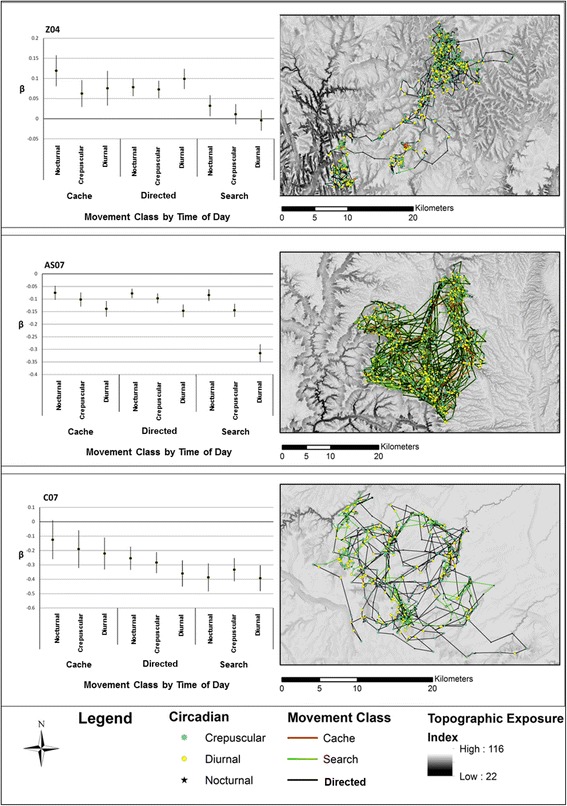
Use of topographic exposure relative to availability by time of day and movement type. Beta coefficients, β, for two males (Z04 and AS07) and a non-reproductive female (C07) show a similar gradual trend in the changes of use from more exposure while at cache sites to less exposure during the day when searching for prey. Topographic availability of each study animal’s home-range is different as shown in the corresponding maps to the right.

**Fig. 7:**
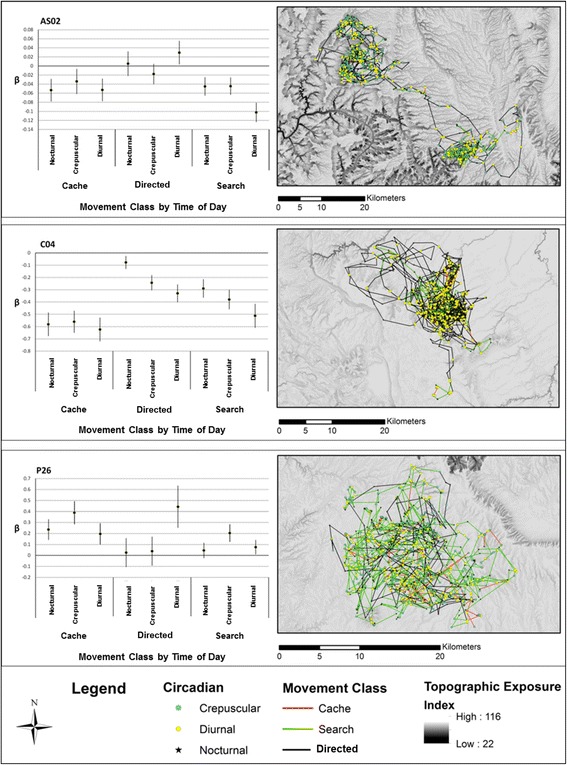
Use of topographic exposure relative to availability by time of day and movement type. Logistic regression beta coefficients, β, for three reproductive females show statistical nonstationarity by time of day and by movement type. Each shows a more positive response to topographic exposure during directed movement, but also displays individualistic variation in relative use.

Relative changes in beta coefficients within an individual suggested statistical nonstationarity related to both circadian activities levels and movement class. The two males, Z04 and AS07, and the non-reproductive female, C07, showed similar relative changes in beta coefficients progressing from high to low (Figure 6 in Appendix). Nocturnal movements while at a cache site showed the most positive relationship with topographic exposure, while diurnal movements during searching had the most negative relationship. Similarly, within a movement class, selection was most negative during diurnal hours, especially for AS07. This relatively negative relationship shows that high movement index values associated with resting activity are inversely related with the topographic exposure index, meaning terrain with low exposure and therefore low visibility is selected when sedentary in the daylight hours. The three reproductive females also displayed statistical nonstationarity (Figure 7 in Appendix), but relative changes in beta coefficients showed a different pattern, with the most relative positive association with topographic exposure occurring during directed movements. This suggests travel occurred on more exposed topography, while cache sites and searching occurred on less exposed topography. Circadian level changes in beta coefficients were variable, suggesting that caring for offspring may complicate the response to topography or other factors may be at play.

## Discussion

Caching foragers present many challenges to the field of movement ecology, due to the differences between their movements relative to the correlated random walk that has been employed so extensively. Because caching animals spend relatively little time searching, and because memory-driven site recursion is common, identifying patterns in movements with step-length and turning-angle alone is difficult and can falsely lead to the conclusion the movement is mostly random. Caching also inherently results in large degrees of behavioral intermittence [39, 57, 58], where movements are made discontinuously due to interspersion of feeding episodes by periods of rest or other activities performed at different locations. Analyses that do not consider behavioral modes or behavioral intermittence tend to bring about the idea of stochasticity and randomness in animal movement because they are incapable of providing explicit mechanistic links between behavior and properties of motion [39].

Progress has been made identifying spatial patterns of wildlife locations such as utilization distributions, and many researchers are starting to identify animal activities and behaviors associated with characteristics of space use [59]. However, attention to the temporal scale of animal movements is still lacking. Efforts to decipher behavioral states in large predators have used arbitrary path-level segmentation based on step-length. For example, Zeller et al. [21] segmented cougar paths based on step-length distances ranging from 12.5 to 200 m to define “resource use”. This definition of use though obscures interpretation of what resources were being selected for, as little movement can occur during periods of resting, feeding, or stalking prey. Though difficult to interpret in practice, Zeller et al.’s (2014) approach was useful for showing that selection of landscape features varies over time and at multiple scales. Similarly, Elliot et al. [28] split GPS telemetry data of African lions into sequential, non-overlapping temporal windows of 30-days and subjectively classified correlograms of step-length into behavioral categories. They found movements also were variable depending on dispersal status, group size, and environmental features. Hidden semi-Markov models have been proposed as an objective way to identify movement modes and behavioral states, able to identify the temporal scale of movements through dwell times. This technique was applied to the Florida panther (*Puma concolor couguar, Kerr*) using step-length and turn-angle metrics and resulted in the identification of three main movement modes: rest, moderate activity, and travel [60]. Van de Kerk et al. found some panthers exhibit highly complex movement, indicated by a large number of potential movement states, but found beyond three states difficult to interpret biologically [60]. Though these studies have recognized the importance of decomposing movement modes in GPS telemetry, they were not able to specifically identify foraging behavior, an important aspect of a species’ ability to occupy an area for periods of time.

Here we explored the utility of creating a movement metric that combines the speed, turning, straightness, and site-fidelity of movements to identify a suite of multiscale behaviors. The approach’s most useful provision is its ability to disentangle circadian activity levels and bouts of feeding-traveling-hunting behavior in a biologically meaningful way. Caching behavior adds a level of complexity when animals seek safe resting sites away from food resources over short time intervals. In the case of cougars, safe resting sites can be environmentally very different than where prey are hunted and cached, making resource selection highly variable in a short temporal window. Pooling movements and therefore activities/behaviors can obscure how differently an animal might respond to its environment depending on its current activity. Ignoring groupings within data can lead to what is known in other disciplines as the “modifiable unit area problem”, the “change of support problem”, the “ecological fallacy”, or “Simpson’s paradox” [61], wherein inference derived across aggregated data can have the opposite response to groupings within.

The approach taken here, in which the unit of analysis is the finest level of observation [23], revealed that the temporal windows of behavioral switches in cougars. The changes in the frequency of switches were also related to seasonal changes in behavior. The ability to adequately identify cache sites, the length of time spent at a cache, and the frequency of feeding over time has important implications for estimates of bioenergetics in cougars and other caching predators. Often resources are not available to investigate every kill site in studies of predator diets using GPS telemetry, and multiple studies have been undertaken to predict cache sites correctly. Franke et al. [62] used hidden Markov models to classify movements and identify sites of kills by wolves in Alberta, Canada, yielding classification accuracy of 74–77% for 24 field investigations. Webb et al. [63] used a GPS clustering technique, similar to the site fidelity measure used here, for wolves to correctly identify 100% of large (moose, elk, horse) prey (*n* = 10) but only 40% of relatively smaller (deer and sheep) prey (*n* = 22) out of 221 total field investigations. Compared to these, our approach had relatively higher classification accuracies in identifying a variety of prey from deer fawns to adult elk. The PII thus appears to be a useful means to provide important information on the foraging movements of caching predators. It also provides a means for locating environmental features contributing to successful predation and denning activities—and therefore identification of key aspects related to predator fitness. Other useful advantages to this approach are its few assumptions and that analysis is not hampered by the proper identification of probability distributions, states, the use of expert opinion, or assigning resistance estimates commonly required by other approaches. Classification of behaviors could use change detection techniques [64] in the PII instead of the expert opinion used here.

Studies demonstrating temporal switching in cyclic behavior have shown the utility of wavelet and frequency transformations [65]. Sine frequency over time was used to model seasonal variation in the level, amplitude, and phase between sea ice characteristics and habitat selection by polar bears [66]. Temporal oscillations of light and temperature, spatiotemporal resource variation (e.g., plant phenology), changing internal physiological states (e.g., hunger or thirst), memory, and dynamic inter- and intraspecific population densities are a few likely contributors to cyclic movements. Resulting movement patterns may have a high degree of temporal correlation at multiple scales and with changing statistical properties relating to scale-specific factors affecting movement [65]. Fourier and wavelet methods, also known as frequency and time–frequency methods, lend themselves well to analyzing the cyclic nature of animal movement and behavior [67, 68]. Here we simply plotted the PII over time to visually identify thresholds in PII values to classify movements into classes of interest and seasonal changes in frequency of behaviors [23], but replacing the typical measures of speed or velocity in these analyses with PII would likely improve frequency analysis of movement. PII also provides a useful means for initial exploration into the temporal structure of movement, as well as a way to identify potential changes in resource selection. This provides a means to segment data for periods of interest such as conducting resource selection evaluations for hunting, migration, or denning behavior.

For cougars PII, a single composite metric, was useful for interpreting movements into behaviors largely because the behaviors of interest are quantified by high and low values of PII. For species whose behaviors of interest result in middle-of-the-range values, PII could be problematic. Middle-of-the-range PII values can be achieved through multiple, behaviorally distinct pathways (e.g., low speeds with moderate turning and site fidelity might produce the same PII as moderate speeds with straight movements and moderate fidelity). For studies where disentangling the mid-PII values we classified as searching into distinct behaviors is important, they may be better served by other analytical approaches. Behavioral change point analysis of movement metrics have proven useful [69] and the use of the temporal heterogeneity in transition probabilities extension of the hidden Markov model framework [70, 71].

Using the movement index to segment data into biologically meaningful path-level analysis showed statistical nonstationarity in how cougars use topographic view-sheds. During a majority of movement classes, the majority of study cougars selected for low topographic exposure during day-time bedding. However, two reproductive females (AS02 and P26) and one male (Z04) were the exception to this during directed movement and actually showed selection towards large view-sheds during the day. Several possible explanations for this behavior exist. Advantages to using these areas may include the ability to detect approaching conspecifics and other threats, observe movement of prey, and for navigation and orientation towards landmarks within a home range. Our hypothesis that cougars use low-exposure areas while sedentary during daylight hours was for the most part supported, but the hypothesis that vantage points and large view-sheds would be selected for during searching for prey was not. We found the exact opposite trend and a strong preference for very low exposure resting sites during bouts of hunting. This behavior might be a result of attempts to go undetected during the day by prey while hunting, since most prey are taken during crepuscular and nocturnal hours [45]. This finding suggests that hunting grounds used by cougars in our study area are conditioned on the availability of low-exposure sites within a distance cougars can travel in a couple of hours.

Several aspects of the observed cougar movements suggest that cognition, memory, and skill level are important factors in how cougars use their landscapes. The migratory path of AS02 (Fig. 5) in the spring (orange path) and fall (brown paths) showed a large degree of overlap, suggesting long-term spatial memory and navigation ability. The repeated directed movement and use of movement corridors by AS07 to cover his range (Figure 6 in Appendix) suggests spatial memory and navigation ability. Caching behavior also demonstrates the use of memory, when cougars bed away from kill sites several kilometers away and then relocate to their cached carcass. Plotting cougar movements over time such as those of AS02 in Fig. 5 suggests cougars are skilled in obtaining prey. Periods defined as searching behavior are relatively short in duration, and a kill site was often observed shortly after a period of fast, directed movement. Thus random movements and area-restricted search in cougars displaying home-range behavior are relatively rare and suggest the use of a modeling framework employing random walks maybe inappropriate for this species.

## Conclusion

Attributing specific behaviors to animal movements based solely on metrics of distance and directionality fails to identify some behaviors. For caching or hoarding foragers, where periods of feeding are interrupted by other activities, distance and directionality measures can be highly variable and uncorrelated over time. Without using additional measures of how observations relate to one another in space and time, patterns in movement can be missed. The spatial resolution and temporal frequency of location measurements now offered by GPS receivers require analytical approaches and metrics that recognize the spatiotemporal structure and nonstationarity of animal resource selection over time. Adding measures of site recursion and fidelity to quantify and identify changes in movement allowed us to more effectively interpret temporal patterns in movements related to circadian routines, foraging activities, seasonal movements, and reproductive behavior. Our movement index, PII, provided a means to segment movement paths in a biologically meaningful way. Quantifying cougar movements in this fashion led to identifying the importance of low exposure topography in the proximity to areas cougars hunt. This conditional relationship has several important implications for predator-prey dynamics and predation risk on the landscape.

## Abbreviations

GPS: Global Positioning System
IACUC: Institutional Animal Care and Use Committee
PII: Path Identification Index
USA: United States of America

## References

[1] Nathan R, Getz WM, Revilla E, Holyoak M, Kadmon R, Saltz D, Smouse PE (2008). A movement ecology paradigm for unifying organismal movement research. Proc Natl Acad Sci.

[2] Holyoak M, Casagrandi R, Nathan R, Revilla E, Spiegel O (2008). Trends and missing parts in the study of movement ecology. Proc Natl Acad Sci.

[3] Gould JL (1987). Landmark learning by honey bees. Anim Behav.

[4] Charnov EL (1976). Optimal foraging, the marginal value theorem. Theor Popul Biol.

[5] Rosenzweig ML, Theory A (1981). Of habitat selection. Ecology.

[6] Johnson DH (1980). The comparison of usage and availability measurements for evaluating resource preference. Ecology.

[7] Kitchin R, Blades M (2002). The cognition of geographic space.

[8] Thinus-Blanc C: Animal spatial cognition: Behavioural and brain approach*.* World Scientific Publishing Co Inc; 1996.

[9] Benhamou S (2007). How many animals really do the levy walk?. Ecology.

[10] Mueller T, Fagan WF, Grimm V (2011). Integrating individual search and navigation behaviors in mechanistic movement models. Theor Ecol.

[11] Hein AM, McKinley SA (2012). Sensing and decision-making in random search. Proc Natl Acad Sci.

[12] Pyke GH (2015). Understanding movements of organisms: it's time to abandon the Lévy foraging hypothesis. Methods Ecol Evol.

[13] Fagan WF, Lewis MA, Auger-Méthé M, Avgar T, Benhamou S, Breed G, LaDage L, Schlägel UE, Tang WW, Papastamatiou YP (2013). Spatial memory and animal movement. Ecol Lett.

[14] Mueller T, Fagan WF (2008). Search and navigation in dynamic environments–from individual behaviors to population distributions. Oikos.

[15] Benhamou S, Riotte-Lambert L (2012). Beyond the utilization distribution: identifying home range areas that are intensively exploited or repeatedly visited. Ecol Model.

[16] Lyons AJ, Turner WC, Getz WM (2013). Home range plus: a space-time characterization of movement over real landscapes. Movement Ecology.

[17] Barraquand F, Benhamou S (2008). Animal movements in heterogeneous landscapes: identifying profitable places and homogeneous movement bouts. Ecology.

[18] Bar-David S, Bar-David I, Cross PC, Ryan SJ, Knechtel CU, Getz WM (2009). Methods for assessing movement path recursion with application to African buffalo in South Africa. Ecology.

[19] Yackulic CB, Blake S, Deem S, Kock M, Uriarte M (2011). One size does not fit all: flexible models are required to understand animal movement across scales. J Anim Ecol.

[20] Getz WM, Saltz D (2008). A framework for generating and analyzing movement paths on ecological landscapes. Proc Natl Acad Sci.

[21] Zeller KA, McGarigal K, Beier P, Cushman SA, Vickers TW, Boyce WM (2014). Sensitivity of landscape resistance estimates based on point selection functions to scale and behavioral state: pumas as a case study. Landsc Ecol.

[22] LaPoint S, Gallery P, Wikelski M, Kays R (2013). Animal behavior, cost-based corridor models, and real corridors. Landsc Ecol.

[23] Benhamou S (2014). Of scales and stationarity in animal movements. Ecol Lett.

[24] Cushman SA, Chase M, Griffin C (2005). Elephants in space and time. Oikos.

[25] Hooten M, Hanks E, Johnson D, Alldredge M (2014). Temporal variation and scale in movement-based resource selection functions. Statistical Methodology.

[26] Postlethwaite CM, Brown P, Dennis TE (2013). A new multi-scale measure for analysing animal movement data. J Theor Biol.

[27] Liu X, Xu N, Jiang A (2015). Tortuosity entropy: a measure of spatial complexity of behavioral changes in animal movement. J Theor Biol.

[28] Elliot NB, Cushman SA, Loveridge AJ, Mtare G, Macdonald DW (2014). Movements vary according to dispersal stage, group size, and rainfall: the case of the African lion. Ecology.

[29] Turchin P (1991). Translating foraging movements in heterogeneous environments into the spatial distribution of foragers. Ecology.

[30] Morales JM, Haydon DT, Frair J, Holsinger KE, Fryxell JM (2004). Extracting more out of relocation data: building movement models as mixtures of random walks. Ecology.

[31] Forester JD, Ives AR, Turner MG, Anderson DP, Fortin D, Beyer HL, Smith DW, Boyce MS (2007). State–space models link elk movement patterns to landscape characteristics in Yellowstone National Park. Ecol Monogr.

[32] Langrock R, King R, Matthiopoulos J, Thomas L, Fortin D, Morales JM (2012). Flexible and practical modeling of animal telemetry data: hidden Markov models and extensions. Ecology.

[33] Jonsen ID, Flemming JM, Myers RA (2005). Robust state–space modeling of animal movement data. Ecology.

[34] Jonsen ID, Myers RA, Flemming JM (2003). Meta-analysis of animal movement using state-space models. Ecology.

[35] Jonsen ID, Myers RA, James MC (2006). Robust hierarchical state–space models reveal diel variation in travel rates of migrating leatherback turtles. J Anim Ecol.

[36] Bischoff-Mattson Z, Mattson D: Effects of simulated mountain lion caching on decomposition of ungulate carcasses. Western North American Naturalist 2009, 69:343–350.

[37] Smith C, Reichman O (1984). The evolution of food caching by birds and mammals. Annu Rev Ecol Syst.

[38] Clayton NS, Bussey TJ, Dickinson A (2003). Can animals recall the past and plan for the future?. Nat Rev Neurosci.

[39] Bartumeus F (2009). Behavioral intermittence, Lévy patterns, and randomness in animal movement. Oikos.

[40] Bell WJ (1990). Central place foraging. *Searching Behaviour.* Springer.

[41] Bryant DM, Turner AK (1982). Central place foraging by swallows (Hirundinidae): the question of load size. Anim Behav.

[42] Chapman CA, Chapman LJ, McLaughlin R (1989). Multiple central place foraging by spider monkeys: travel consequences of using many sleeping sites. Oecologia.

[43] Cornélis D, Benhamou S, Janeau G, Morellet N, Ouedraogo M, De Visscher M-N (2011). Spatiotemporal dynamics of forage and water resources shape space use of west African savanna buffaloes. J Mammal.

[44] Milner-Gulland E, Fryxell JM, Sinclair AR. Animal migration: a synthesis: Oxford University Press; 2011.

[45] Mattson DJ: Mountain Lions of the Flagstaff Uplands: 2003-2006 Progress Report 2007.

[46] Benhamou S (2004). How to reliably estimate the tortuosity of an animal's path:: straightness, sinuosity, or fractal dimension?. J Theor Biol.

[47] Pierce BM, Bleich VC, Chetkiewicz C-LB, Wehausen JD: Timing of feeding bouts of mountain lions. J Mammal 1998, 79:222–226.

[48] Bovet P, Benhamou S (1988). Spatial analysis of animals' movements using a correlated random walk model. J Theor Biol.

[49] Fauchald P, Tveraa T (2003). Using first-passage time in the analysis of area-restricted search and habitat selection. Ecology.

[50] Tél T, Gruiz M. Chaotic dynamics: an introduction based on classical mechanics: Cambridge University Press; 2006.

[51] Knell AS, Codling EA (2012). Classifying area-restricted search (ARS) using a partial sum approach. Theor Ecol.

[52] Knopff KH, Knopff AA, Warren MB, Boyce MS (2009). Evaluating global positioning system telemetry techniques for estimating cougar predation parameters. J Wildl Manag.

[53] Manly B, McDonald L, Thomas D, McDonald TL, Erickson WP. Resource selection by animals: statistical design and analysis for field studies. Springer Science & Business Media. 2007;

[54] Burdett CL, Crooks KR, Theobald DM, Wilson KR, Boydston EE, Lyren LM, Fisher RN, Vickers TW, Morrison SA, Boyce WM (2010). Interfacing models of wildlife habitat and human development to predict the future distribution of puma habitat. Ecosphere.

[55] Yokoyama R, Shirasawa M, Pike RJ (2002). Visualizing topography by openness: a new application of image processing to digital elevation models. Photogramm Eng Remote Sens.

[56] Ironside KE, Mattson D, Choate D, Stoner D, Arundel TR, Hansen J, Theimer T, Holton B, Jansen B, Sexton JO, et al: Variable Terrestrial GPS Telemetry Detection Rates: Parts 1 - 7— Data: U.S. Geological Survey; 2015. https://doi.org/10.5066/F7PG1PT2.

[57] O’brien WJ, Browman HI, Evans BI (1990). Search strategies of foraging animals. Am Sci.

[58] Kramer DL, McLaughlin RL (2001). The behavioral ecology of intermittent locomotion 1. Am Zool.

[59] Benhamou S, Cornélis D (2010). Incorporating movement behavior and barriers to improve kernel home range space use estimates. J Wildl Manag.

[60] Kerk M, Onorato DP, Criffield MA, Bolker BM, Augustine BC, McKinley SA, Oli MK (2015). Hidden semi-Markov models reveal multiphasic movement of the endangered Florida Panther. J Anim Ecol.

[61] Clark JS. Models for ecological data: an introduction: Princeton university press Princeton; 2007.

[62] Franke A, Caelli T, Kuzyk G, Hudson RJ (2006). Prediction of wolf (Canis Lupus) kill-sites using hidden Markov models. Ecol Model.

[63] Webb NF, Hebblewhite M, Merrill EH (2008). Statistical methods for identifying wolf kill sites using global positioning system locations. J Wildl Manag.

[64] Sonderegger DL, Wang H, Clements WH, Noon BR (2009). Using SiZer to detect thresholds in ecological data. Front Ecol Environ.

[65] Polansky L, Wittemyer G, Cross PC, Tambling CJ, Getz WM (2010). From moonlight to movement and synchronized randomness: Fourier and wavelet analyses of animal location time series data. Ecology.

[66] Ferguson SH, Taylor MK, Messier F (2000). Influence of sea ice dynamics on habitat selection by polar bears. Ecology.

[67] Wittemyer G, Polansky L, Douglas-Hamilton I, Getz WM (2008). Disentangling the effects of forage, social rank, and risk on movement autocorrelation of elephants using Fourier and wavelet analyses. Proc Natl Acad Sci.

[68] Riotte-Lambert L, Benhamou S, Chamaillé-Jammes S (2013). Periodicity analysis of movement recursions. J Theor Biol.

[69] Gurarie E, Andrews RD, Laidre KL (2009). A novel method for identifying behavioural changes in animal movement data. Ecol Lett.

[70] McClintock BT (2017). Michelot T: momentuHMM: R package for analysis of telemetry data using generalized multivariate hidden Markov models of animal movement.

[71] Li M, Bolker BM (2017). Incorporating periodic variability in hidden Markov models for animal movement. Movement Ecology.

